# Genetic variation along an altitudinal gradient in the *Phytophthora infestans* effector gene *Pi02860*

**DOI:** 10.3389/fmicb.2022.972928

**Published:** 2022-09-08

**Authors:** Li-Na Yang, Haibing Ouyang, Oswald Nkurikiyimfura, Hanmei Fang, Abdul Waheed, Wenyang Li, Yan-Ping Wang, Jiasui Zhan

**Affiliations:** ^1^Fujian Key Laboratory on Conservation and Sustainable Utilization of Marine Biodiversity, Fuzhou Institute of Oceanography, Minjiang University, Fuzhou, China; ^2^Department of Plant Pathology, Nanjing Agricultural University, Nanjing, China; ^3^Institute of Plant Pathology, Fujian Agriculture and Forestry University, Fuzhou, China; ^4^College of Chemistry and Life Sciences, Sichuan Provincial Key Laboratory for Development and Utilization of Characteristic Horticultural Biological Resources, Chengdu Normal University, Chengdu, China; ^5^Department of Forest Mycology and Plant Pathology, Swedish University of Agricultural Sciences, Uppsala, Sweden

**Keywords:** adaptation, agriculture, population genetics, plant pathogen, virulence factor, climate change, natural selection, molecular evolution

## Abstract

Effector genes, together with climatic and other environmental factors, play multifaceted roles in the development of plant diseases. Understanding the role of environmental factors, particularly climate conditions affecting the evolution of effector genes, is important for predicting the long-term value of the genes in controlling agricultural diseases. Here, we collected *Phytophthora infestans* populations from five locations along a mountainous hill in China and sequenced the effector gene *Pi02860* from >300 isolates. To minimize the influence of other ecological factors, isolates were sampled from the same potato cultivar on the same day. We also expressed the gene to visualise its cellular location, assayed its pathogenicity and evaluated its response to experimental temperatures. We found that *Pi02860* exhibited moderate genetic variation at the nucleotide level which was mainly generated by point mutation. The mutations did not change the cellular location of the effector gene but significantly modified the fitness of *P. infestans*. Genetic variation and pathogenicity of the effector gene were positively associated with the altitude of sample sites, possibly due to increased mutation rate induced by the vertical distribution of environmental factors such as UV radiation and temperature. We further found that *Pi02860* expression was regulated by experimental temperature with reduced expression as experimental temperature increased. Together, these results indicate that UV radiation and temperature are important environmental factors regulating the evolution of effector genes and provide us with considerable insight as to their future sustainable action under climate and other environmental change.

## Introduction

Plant diseases have deleterious effects on ecological integrity and agricultural productivity, greatly threatening natural sustainability and global food security. For example, damage caused by plant diseases such as chestnut blight and Dutch elm disease to primary and secondary forests not only reduces species richness and the associated ecological services (Martín et al., 2013) but has also restructured ecological landscapes in North America and Europe (Raum, 2020). From a social-economic perspective, 13–20% of global agricultural harvest (Burdon et al., 2020) corresponding to ~US$220 billion is lost due to plant disease epidemics annually. Moreover, it is of great concern that the ecological and societal impact of plant diseases may be further aggravated by global climate change (Cavicchioli et al., 2019).

Effector proteins, either directly secreted into the apoplast or translocated into host cells, play critical roles in the antagonistic interaction between hosts and pathogens (Lo Presti et al., 2015; Wang et al., 2017). They are mainly involved in the regulation of the host’s immunity system but also take part in nutrient uptake of pathogens from hosts (Uhse and Djamei, 2018). To broaden their host range and increase their successful colonization and reproduction, both expression level and profile of pathogen effector genes are strictly regulated during antagonistic interactions, and show waves of concerted expression at diverse stages of infection and reproduction according to the surrounding environment and genetic background of hosts (Wang et al., 2011; Soyer et al., 2014; Ren et al., 2020). For example, some effector genes are upregulated to promote host susceptibility (Boevink et al., 2016) and subdue the host immune system (Turnbull et al., 2017), while others are down regulated to avoid host recognition (Lo Presti et al., 2015).

It has been found that plant pathogens encode a large number of effectors in order to ensure successful colonization and reproduction by suppressing host defence immunity (Franceschetti et al., 2017). The specialized location of many effector genes in the gene-sparse, repeat-rich regions of the genome provide them with unique niches for quick evolution (Haas et al., 2009; Raffaele and Kamoun, 2012; Dong et al., 2015) and hence the ability to defeat host defence immunity involved in gene-for-gene interactions (Fry, 2008; Vleeshouwers et al., 2011). The arm race between host resistance genes and pathogen effector genes is particularly intense in agricultural ecosystems due to the strong directional selection generated by farming practices associated with crop intensification and genetic homogenization (Zhan et al., 2014, 2015). It is widely documented that pathogens in agricultural ecosystems are equipped with an array of mutation mechanisms to drive their escape from host immunity. For example, *Phytophthora infestans Avr1* and *Avr4* were removed from the effector repertoire by pseudogenization to escape the corresponding R1 and R4 detection in host (van Poppel et al., 2008; Shen et al., 2021; Waheed et al., 2021). *Phytophthora infestans Avr2* escapes host immunity by protein disordering through which shifts the effector from an avirulent type to virulent type (Yang et al., 2020). On the other hand, *P. infestans Avr3a* overcomes R3a recognition by potato crop through point mutations in two residues (K^80^I^103^ to E^80^M^103^) where protein (effector)-protein (receptor) interactions take place (Boutemy et al., 2011; Yaeno et al., 2011).

As an important part of the disease triangle, climatic conditions can regulate the growth and development of pathogens and hosts as well as their interactions (Juroszek et al., 2020). In addition to host resistance and the biology of pathogens, recent studies have shown that the evolution and performance of effector genes are also affected by climate conditions. For example, the effector function of *P. syringae AvrRpt2* to *Arabidopsis RPS2* resistance disappeared when plants were grown for 3 weeks under 28°C (Wang et al., 2009). The strong association of annual mean temperature in the collection sites with nucleotide diversity and/or population differentiation of *Avr1* (Shen et al., 2021), *Avr2* (Yang et al., 2020), *Avr3a* (Yang et al., 2018) and *Avr4* (Waheed et al., 2021) in *P. infestans* further supports that local air temperature could influence the evolution of effector genes.

Our knowledge about the evolution of effector genes is fragmented. Most research on this part of genome has focused on molecular characterization and functional analysis. Population genetic analysis of the spatial distribution of effector genes to understand their evolutionary history, mechanisms and future trajectory is limited. Climate changes in the planet have accelerated in the past decades due to the human activities including agricultural production. It is predicted that average air temperature would increase ~5°C by the end of the century (Tollefson, 2020). UV radiation, another important determinant of disease epidemics and pathogen evolution, is also expected to increase associated with the current wave of climate change due to the thinning ozone layer associated with emission of some industrial gases (Bais et al., 2015). Knowledge of how and to what extent these climate and climate related factors may impact the evolution and performance of effector genes is important to understand future ecological and agricultural sustainability under changing climates.

In this manuscript, we used a population genetic approach to study the evolution of *Pi02860*, one of confirmed effector gene in the interaction of *P. infestans* with potato host (Yang et al., 2016b) and how ecological factors may influence its evolution. *P. infestans* is one of the most widespread oomycete plant pathogens causing late blight disease of potato and tomato. Genome analysis revealed that *P. infestans* codes many effectors that are delivered into plant cells during infection where they interfere with host immunity (Haas et al., 2009). *Pi02860* is one of the effectors that are specifically upregulated at ~2 days after infecting potato plants (Yang et al., 2016b). It is found that this effector significantly suppressed the cell death triggered by pathogen-associated molecular pattern INF1 and strongly enhanced the colonization ability of *P. infestans* (Yang et al., 2016b). It interacted with the host susceptibility protein NRL1 and enhanced the assembly of NRL1 with SWAP70, a positive regulator of immunity, to promote proteasome-mediated degradation of the latter and, thus, suppress immunity (Yang et al., 2016b; He et al., 2018).

The specific objectives of the study were to answer four questions: (i) what is the genetic variation of *Pi02860*? (ii) How is the genetic diversity generated? (iii) What are the pathogenicity and cellular consequences of the observed mutations? and (iv) how may climatic or climate-related factors such as UV radiation and air temperature affect the performance and spatial distribution of genetic variation in the effector gene? Altitude change provides a strong reflection of climatic gradients across short spatial distances, offering a great opportunity to study the influence of climate conditions particularly UV light and temperature on the biology and ecology of species (Körner, 2007). For example, UV intensity increases 10–20% and temperature decreases ~6.5°C for every 1,000 m elevation. To achieve our objectives, we sampled >300 isolates from five altitudinal sites along a mountainous hill and sequenced the effector gene *Pi02860*. Genetic variation, mechanisms responsible for the generation of this variation, cellular localization, altitudinal distribution and expression of the effector gene under different experimental temperatures were analysed. A total of 21 nucleotide haplotypes mainly generated by point mutations were found in the collections. The spatial distribution of the effector gene was associated with the altitude of collection sites and the expression of the effector was strongly regulated by experimental temperatures.

## Materials and methods

### *Phytophthora infestans* collections

*Phytophthora infestans* isolates were collected in a single day during the 2016 late blight epidemic season from a single potato cultivar (Hui-2) grown in five fields along along a mountainous hill located at Huize, Yunnan. The altitudes of the five fields ranged from 1,976 to 2,677 m and were designated as A–E from lower to higher altitude, respectively (Table 1). One hundred *P. infestans* infected plants each at least 1-m apart were selected from each field and one infected leaf was sampled from the upper canopy of each plant. The sampled leaves were transferred to the laboratory within 24 h for *P. infestans* isolation. A single isolate was secured from each sample, resulting in a collection of a total of 354 isolates with 59–87 isolates originating from each of the five fields. The isolates were purified three times by sequential transfers of a single sporangium to a fresh rye B plate supplemented with ampicillin (100 μg/ml) and rifampicin (50 μg/ml). Detailed information on the sample collection and *P. infestans* isolation can be found in our previous publications (Zhu et al., 2015; Yang et al., 2016a).

**Table 1:** Sample size, and genetic variation of *Phytophthora infestans Pi02860* sequences collected from five altitudinal locations.

Population	Size	Altitude (m)	S	H	PH	HD	Π
A	65	1,976	3	5	1	0.5817	0.00165
B	86	2,124	4	5	1	0.5499	0.00165
C	59	2,471	7	6	3	0.6791	0.00232
D	77	2,591	23	10	6	0.6702	0.00228
E	67	2,677	8	10	6	0.6739	0.00274
Total	354		35	21	17	0.6240	0.00231

S, number of segregating sites; H, number of haplotypes; PH, number of private haplotypes, i.e., which only present in one population; HD, haplotype diversity; π, nucleotide diversity.

### *Pi02860* sequencing

*Phytophthora infestans* isolates were cultured on rye B agar at 18°C in the dark. Mycelia (~100 mg) were harvested after 15 days cultivation, transferred into sterile, 2 ml centrifuge tubes and lyophilized with a vacuum freeze dryer (Alpha1-2, Christ, Germany). The lyophilized mycelia were ground to powder with a mixer mill (MM400, Retsch, Germany). Total DNA was extracted using a Plant gDNA Miniprep Kit (GD 2611, Biomiga, China) according to the manufacturer’s instructions. The genomic DNA was suspended in 50 μl of ultrapure water and stored at −20°C until use.

A pair of *Pi02860* specific primers (For: 5′-ACTCACCGTCACCCTCATTC-3′ and Rev: 5′-AACTTTGACTCCGACCGTTG-3′) were designed according to the conserved upstream and downstream of the reference sequence (PITG_02860) downloaded from NCBI and used to amplify the *Pi02860* effector gene. PCR reactions were carried out in a 25 μl reaction volume using a thermal cycler (Applied Biosystems 2720). The reaction cocktail contained 1 × PCR buffer, 0.2 mM dNTPs, 1 unit of TransStart KD Plus DNA Polymerase, 0.2 μM of primers and 20 ng of template DNA. PCR amplification was started with an initial denaturation step of 94°C for 2 min, followed by 30 cycles of amplification at 95°C for 30 s, annealing at 55°C for 30 s, extension at 72°C for 50 s, and ended with a further extension cycle at 72°C for 10 min. The PCR products were separated by 1% agarose gel electrophoresis and sent to Sangon Biotech company for sequencing using an ABI3730 automated DNA sequencer (Applied Bio-Systems, United States).

### Plant preparation

*Nicotiana benthamiana* and a universal susceptible potato variety Desiree were grown in a greenhouse at 20°C in 60% humidity. The greenhouse was supplemented with 16 h-light at the intensity of 120–150 μmol m^−2^ s^−1^, nutrient and water were supplied at necessary. *Nicotiana benthamiana* plants were used for agroinfiltration at 4–5 weeks old and potato leaves at 5–6 weeks old were used for *P. infestans* infection analysis.

### Vector construction

After removing those with mutations in the start codon or translating to the same isoforms, 16 *Pi02860* haplotypes without a signal peptide were amplified from genomic DNAs of corresponding *P. infestans* isolates. PCR products were ligated into pEarlyGate 104 (N-terminal GFP tag) using a Vazyme ClonExpressII One Step Cloning Kit, and then transformed into *Agrobacterium tumefaciens* strain AGL1. The Avr3a and INF1 constructs with N-terminal GFP tag were kindly provided by Dr. Qin He from Huazhong Agricultural University, Wuhan, China.

### Agroinfiltration, infection assays, HR suppression and cellular localization

*Agrobacterium tumefaciens* strain AGL1 transformed with the vector constructed above was grown overnight in LB medium containing selective antibiotics at 28°C, pelleted, resuspended in infiltration buffer (10 mM MES, 10 mM MgCl_2_), and adjusted to the required optical density measured at 600 nm (OD_600_) before being infiltrated into *N. benthamiana* leaves (generally 0.005–0.01 for cellular localization purposes, 0.25 for infection assays, and 0.5 for hypersensitive reaction assays). For co-expression, agrobacterial cultures carrying the appropriate vector constructs were mixed prior to infiltration.

*Phytophthora infestans* strain 88,069 (Yang et al., 2021) was used for plant infection. It was cultured on rye agar at 18°C for 2 weeks before sporangia collection. The inoculum concentration was adjusted to 40,000 sporangia per ml, and 10 μl droplets were inoculated onto the abaxial side of leaves of intact *N. benthamiana* plants which were transiently expressed with *Pi02860* haplotypes in sealed boxes. The average lesion diameter was pictured at 6–8 days after inoculation (DAIs).

Cellular localization of *Pi02860* effectors was investigated on *N. benthamiana* plants by confocal imaging. In this analysis, *N. benthamiana* cells were imaged at 2 DAIs using a Nikon Ti-E inverted microscope with Plan Apo 40X/0.95 dry objective. GFP was excited by 488-nm light shed from an argon laser, and 500–530 nm emissions were captured. To minimize potential artifacts of ectopic protein expression, only single optical images from leaf cells expressing low levels of the protein fusions were collected and analysed.

### Analyses of thermal-mediated *Pi02860* expression

Fifteen *P. infestans* isolates with different *Pi02860* haplotypes were inoculated onto the detached leaves of the universal susceptible potato cultivar at low (10°C), near optimum (18°C) (near optimum) and high (25°C) temperature of the pathogen infection (Yang et al., 2016a). Three 6-mm leaf disks were excised from the inoculation sites 2 DAIs. RNA was extracted from the leaf disks using the *EasyPure* plant RNA Kits (TransGen Biotech) according to the manufacturer’s instructions. RNA was quantified using a Nanodrop 1000 (Thermo Scientific) and cDNA was synthesized using TransScript One-Step gDNA Removal and cDNA Synthesis SuperMix (TransGen Biotech) according to the manufacturer’s instructions. QRT-PCR was performed using TransStart Top Green qPCR SuperMix (TransGen Biotech) and run on a QuantStudio 5 Real-Time PCR Instrument (Applied Biosystems) using QuantStudio Design & Analysis software v1.4.2 with specific primers (For: GTGTCGCCTGGTCTAATCC, Rev: TTCTCTCTTCATTGGCTTCG). The expression of the effector gene was analyzed using the ΔΔCt method (McLellan et al., 2013) normalized with the housekeeping gene ActinA.

### Data analyses

Before analysis, all *Pi02860* nucleotide sequences were visually assessed to remove potential artifacts (Yang et al., 2013). *Pi02860* isoforms were deduced from nucleotide sequences and multiple sequence alignments were performed by the ClustalW implemented in MEGA 7.0.21 (Kumar et al., 2016). Nucleotide haplotypes were reconstructed with the PHASRE algorithm implemented in DnaSP 5.10 (Librado and Rozas, 2009). The DnaSP 5.10 program was also used to estimate haplotype diversity, nucleotide diversity, the rates of non-synonymous substitutions and synonymous substitutions. Haplotype and nucleotide diversities were estimated for each of the five populations (elevations) as well as the combined population by pooling the sequences for individual populations. A median joining (MJ) network illustrating genealogical relationships among haplotypes was generated using PopART 1.7 (Leigh and Bryant, 2015). The population scaled recombination rates were assessed by INTERVAL program implemented in LDHAT package at the interface of RDP4 (McVean et al., 2002, 2004). The mean lesion size (INF1 cell death suppression) in each population was calculated with the formula as follows:


$$Y=\sum \left(w_{i}p_{i}\right)$$

where *w_i_* and *p_i_* represent the frequency and the observed lesion size (INF1 cell death suppression) of haplotype *i* and Y is the mean lesion size (INF1 cell death suppression) in the population. Associations of sequence diversity with biological characteristics of the effector gene and altitude were evaluated by Pearson’s correlation (Lawrence and Lin, 1989). Duncan’s multiple range and LSD tests (Ott and Longnecker, 2015) were applied to determine the differences in lesion size, INF1 induced cell death and gene expression using the SAS software. A Chi-square test for haplotype homogeneity among different altitudes was conducted by SPSS 19.0.

## Results

### Sequence variation in *Pi02860* effector gene

**Figure 1:**
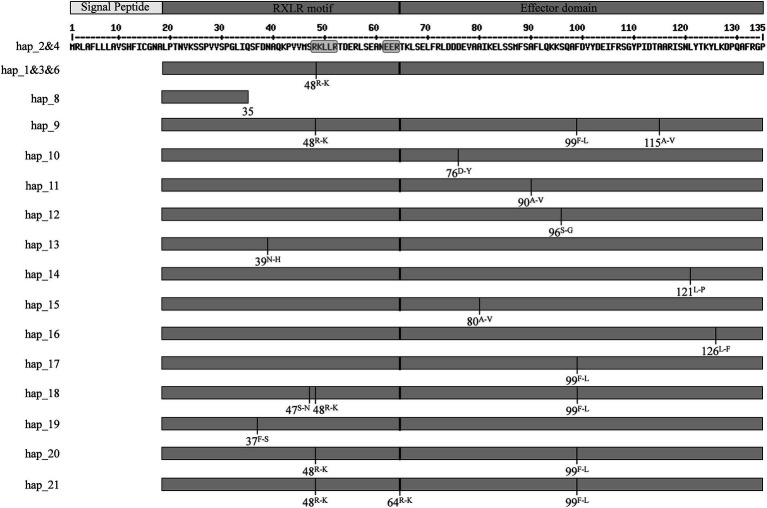
Sketch showing the *Pi02860* structural domain and the mutation position in each of nucleotide haplotypes. Hap_5 and Hap_7 were removed due to a mutation in start codon.

A total of 35 variable sites were detected in the 354 full nucleotide sequences, representing 3–23 sites from each of the five populations (Table 1). These variable sites formed 21 nucleotide haplotypes. Nearly all sequence variations were generated by point mutations (Supplementary Table 1). Besides that, an early termination stop codon was generated by point mutation at the 107^th^ nucleotide in Hap_8, causing truncation within the RXLR motif (Figure 1; Supplementary Table 1). Pseudo genes generated by deletions in the start codon were found in two isolates (Supplementary Table 1).

### Altitudinal distribution of *Pi02860* gene

**Figure 2:**
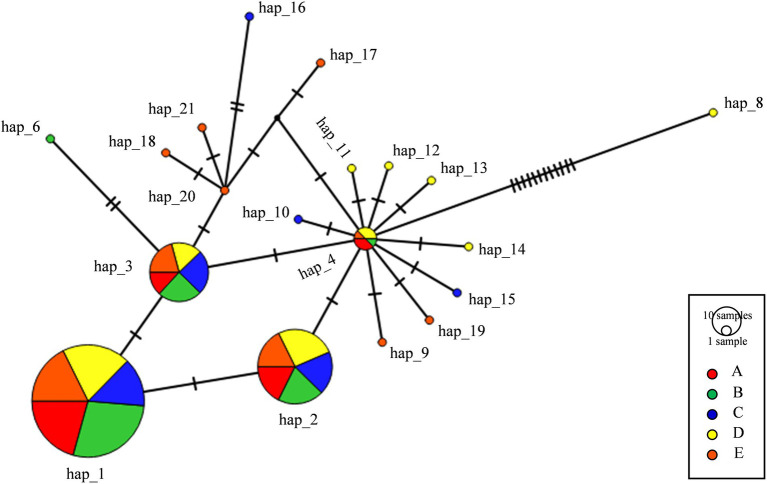
Haplotype network of *Pi02860* gene. Nucleotide haplotypes are named with Hap followed by a number. Size of the circles indicates haplotype frequencies in the population. Colors correspond to the respective populations. Each tick mark represents a step of nucleotide substitution. Hap_5 and Hap_7 have a mutation in start codon and were removed from haplotype network construction.

Among the 21 nucleotide haplotypes, Hap_1 to Hap_4 were major haplotypes with a frequency ranging from 0.00 to 62.79% in individual populations (Supplementary Table 2) and ranging from 2.26 to 54.24% in the combined population. Hap_1, Hap_2, and Hap_3 were observed in all five populations while Hap_4, was observed in four of the five populations (Figure 2). Other haplotypes were detected only once and were private to each population (Table 1; Figure 2). The pathogen populations from different altitudes varied significantly in the frequency of both nucleotide haplotypes (Supplementary Table 2) and isoforms (Supplementary Table 3) by the homogeneity test, with a value of *p* of 0.009 and 0.044, respectively. The haplotype diversity of nucleotide sequences in the five populations ranged from 0.5499 to 0.6791 with a mean of 0.6240 when sequences from the five sites were combined. Nucleotide diversity in the populations ranged from 0.00165 to 0.00274 with a grand mean of 0.00231 (Table 1). Populations collected from the lowest altitudes (A and B) had the least number of haplotypes and lowest haplotype nucleotide diversity while population E from the highest altitude displayed the greatest number of haplotypes and highest nucleotide diversity (Table 1).

### Haplotype network

Hap_4 was identical to T30-4, the reference sequence. Hap_1, Hap_2 and Hap_3 had one SNP in 30^T-C^ or 143^G-A^ compared to the reference sequence (Supplementary Table 1). Due to some synonymous mutations, Hap_1 and Hap_3 were translated to the same amino acid isoform, accounting for 68.93% of the combined population while Hap_2 and Hap_4 were translated to another isoform, accounting for 26.27% of the combined population (Supplementary Table 3). All dominant haplotypes were one or two steps away from each other except Hap_8 which has an early termination at the 36th amino acid position and some rare haplotypes (Figure 2).

### Association between sequence variation and altitude

**Figure 3:**
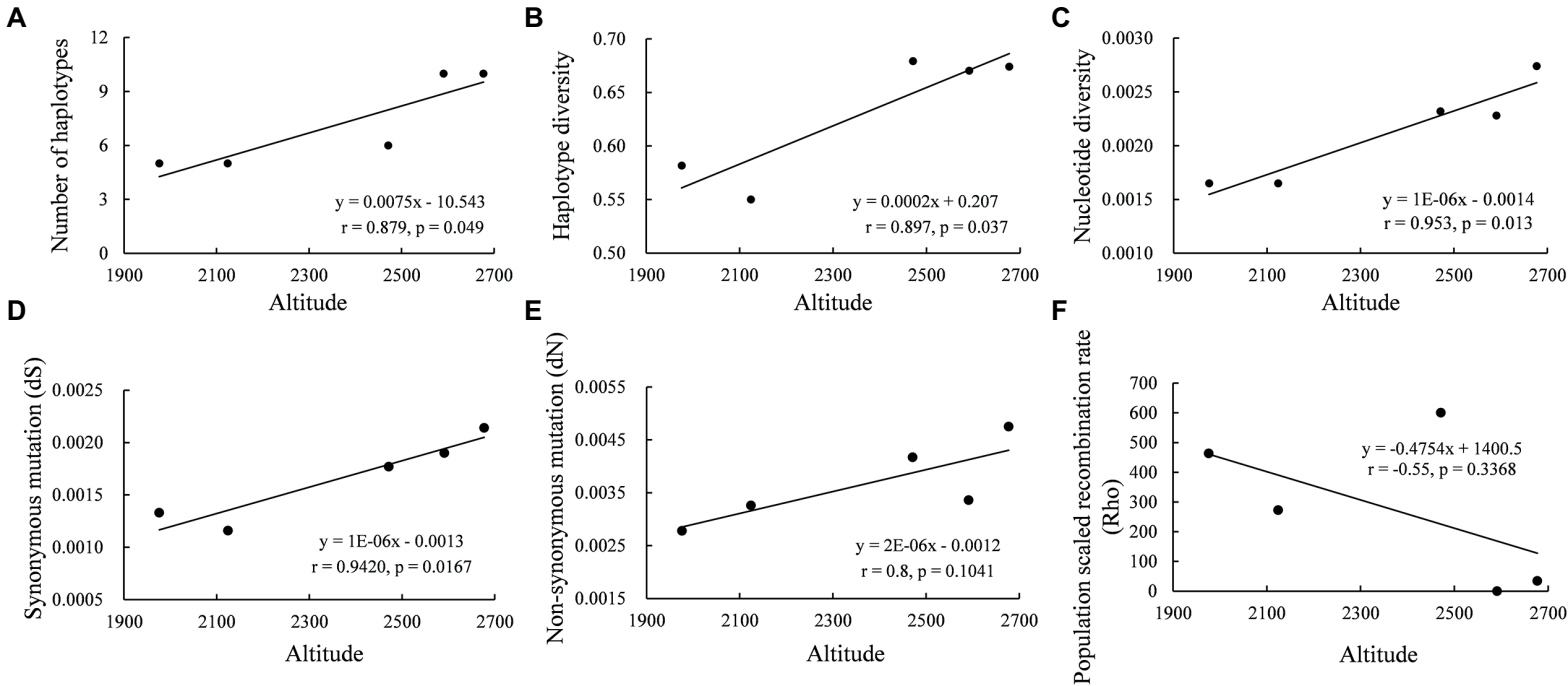
Correlations between the altitude of the collection sites of the pathogen and the genetic variation of *Pi02860* gene: **(A)** the number of haplotypes; **(B)** haplotype diversity; **(C)** nucleotide diversity; **(D)** synonymous mutation; **(E)** nonsynonymous mutation; **(F)** population scaled recombination rate of *Pi02860* and altitude.

Haplotype richness of *Pi02860* was positively associated with altitude (*p* = 0.049, Figure 3A). The haplotype diversity and nucleotide diversity of the effector gene were also positively correlated with altitude, with a value of *p* of 0.037 and 0.013, respectively (Figures 3B,C). Both synonymous mutation (dS) and non-synonymous mutation (dN) rates were positively correlated with altitude, but only the correlation with synonymous mutation was significant (Figures 3D,E). Population recombination rate was negatively correlated with altitude but was not significant (Figure 3F).

### The subcellular localization of *Pi02860* haplotypes

**Figure 4:**
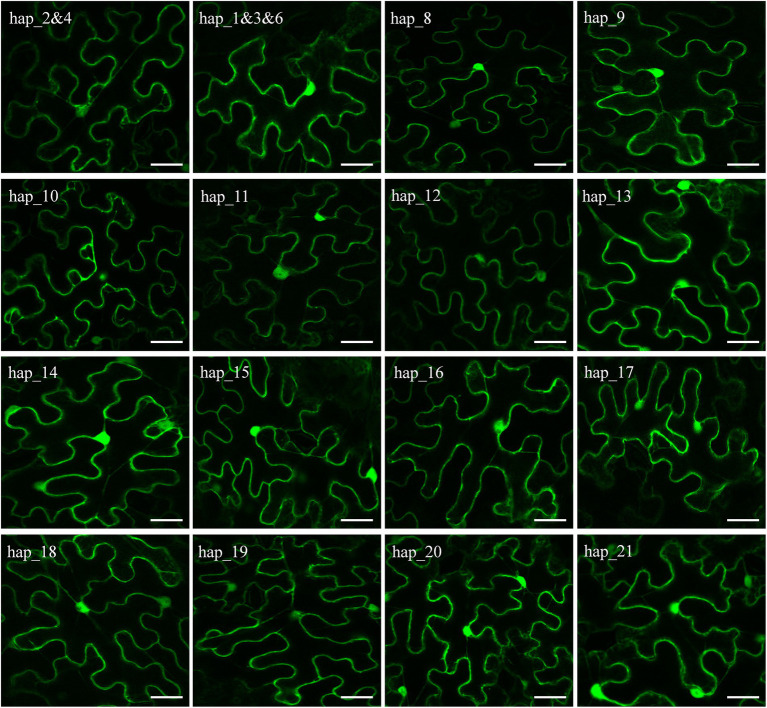
Confocal images showing that GFP-*Pi02860* effectors are localized in the cytoplasm and nucleus. Scale bars are 20 μm.

Sixteen vectors were constructed to transiently express *Pi02860* haplotypes without a signal peptide in *N. benthamiana* for subcellular localization analysis. Two of the 21 haplotypes which are considered as pseudogenes due to a point mutation in the start codon were removed from the cellular localization analysis. Three haplotypes (Hap_1, Hap_3, and Hap_6) translate into one identical isoform and two haplotypes (Hap_2 and Hap_4) translate into another identical isoform (Figure 1). Only one vector was constructed for each of these two isoforms. Confocal images showed that non-synonymous mutation at these nucleotide sites (37th, 39th, 47th, 48th, 64th, 76th, 80th, 90th, 96th, 99th, 115th, 121st and 126th) did not change the subcellular localization of the *Pi02860* effector. All GFP-tagged *Pi02860* haplotypes were found to localize in the cytoplasm and nucleus (Figure 4).

### Virulence analysis of *Pi02860* haplotypes

**Figure 5:**
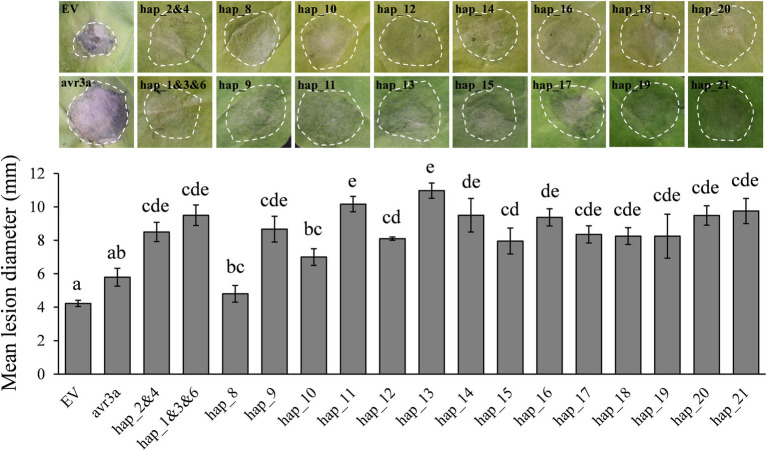
Transient overexpression of *Pi02860* haplotypes in *N. benthamiana* increases *P. infestans* lesion diameter to various degrees compared with free GFP. Bottom: statistical test; Top: representative images of pathogen expression. EV and avr3a are negative and positive controls, respectively. Hap_1 to Hap_21 are the codes of 21 haplotypes detected. Hap_2 and 4 are deduced into the same isoform and Hap_1, 3 and 6 are deduced into another the same isoform, thus only one vector was constructed for each of the two haplotype groups. Results shown are derived from the mean of at least three independent biological replicates.

**Figure 6:**
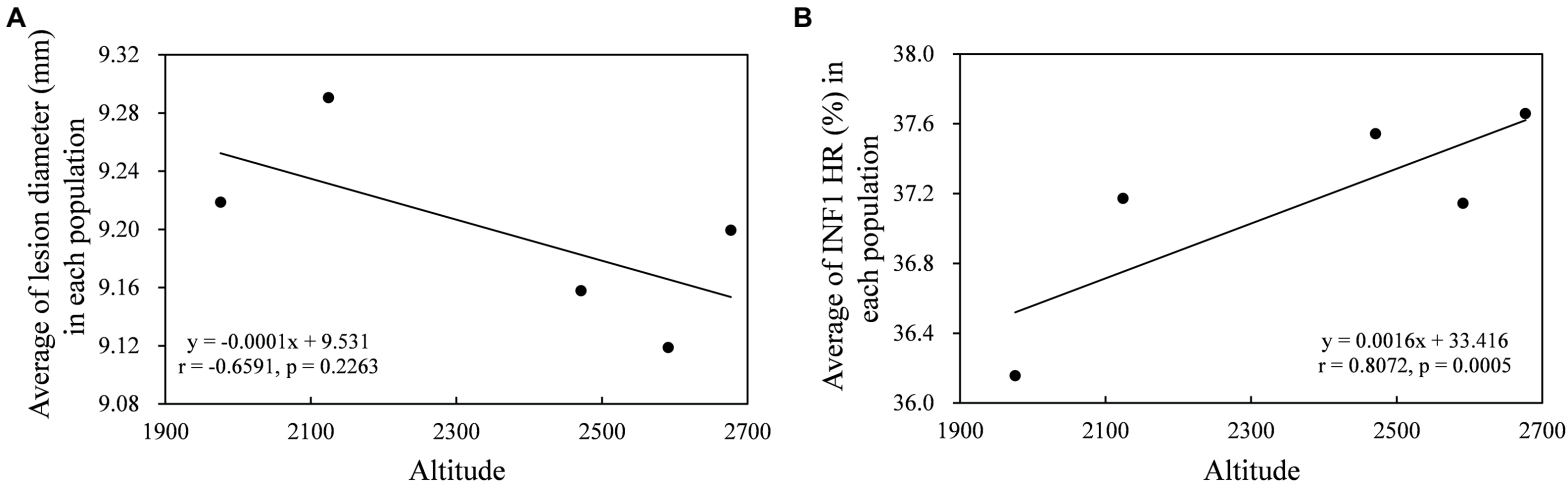
Correlation between the altitude of the collection sites and lesion size **(A)** and INF1 suppression **(B)** of pathogen population.

GFP-tagged expression revealed all *Pi02860* haplotypes including the truncated haplotype promoted the lesion size of co-expressed host *N. benthamiana* and there was also a significant difference in pathogenicity among the effector haplotypes (Figure 5). Hap_2 and Hap_4, which translate into isoform identical to the wild type *Pi02860* effector (T30-4), produced lesions larger than Hap_8, *Avr3a* and the negative control (EV). Hap_1, Hap_3 and Hap_6 which have one SNP in 48^K-R^ compared to the wild type *Pi02860* effector, as well as other haplotypes except Hap_8, produced larger lesions than Hap_2 and Hap _4 did. Hap_8, which has a terminal truncation starting at the 36^th^ amino acid position, produced significantly smaller lesions than other haplotypes including *Avr3a* (Figure 5). The altitude of collection site was negatively associated with the lesion size of the pathogen but was not significant (Figure 6A).

**Figure 7:**
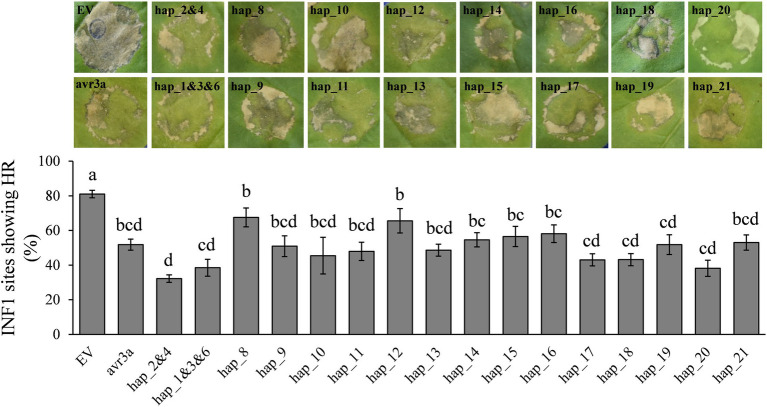
Transient overexpression of GFP-*Pi02860* haplotypes in *N. benthamiana* suppresses the HR (hypersensitive response) triggered by the elicitin INF1. Top: images of pathogen expression: Bottom: statistical test. EV and avr3a are negative and positive controls, respectively. Hap_1 to Hap_21 are the codes of 21 haplotypes detected. Hap_2 and 4 are deduced into the same isoform and Hap_1, 3 and 6 are deduced into another the same isoform, thus only one vector was constructed for each of the two haplotype groups. Results shown are derived from the mean of at least three independent biological replicates.

*Pi02860* haplotypes varied significantly in suppressing INF1 cell death (Figure 7). Although there was no significantly correlation, the suppression of INF1 cell death caused by effector haplotypes largely mirrored the lesion sizes they produced on *N. benthamiana* (Figures 5, 7). In this expression analysis, Hap_2 and Hap _4 had the strongest suppression on cell death elicited by INF1, followed by Hap_1 Hap _3 and Hap _6, the most dominant *Pi02860* effector haplotypes. Hap_8 had nearly no effect on INF1 cell death suppression. The average of INF1 cell death suppression in the *P. infestans* populations was positively and significantly correlated with altitude (Figure 6B).

### Thermal-mediated expression polymorphisms in *Pi02860* effector gene

**Figure 8:**
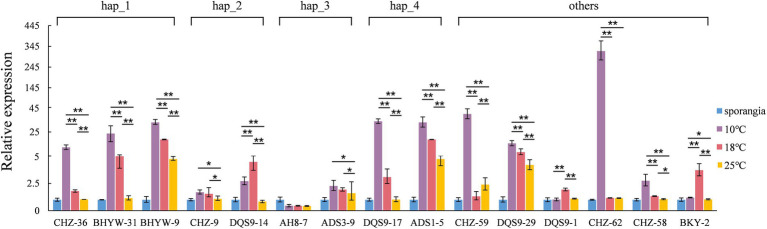
The expression of *Pi02860* haplotypes under 10, 18 and 25°C.

QRT-PCR analysis of *Pi02860* expression at 2DAIs on susceptible potato plants showed that the transcription accumulation varied substantially among isolates with different effector haplotypes and among experimental temperatures (Figure 8). In all but isolate DQS9-14 of Hap_2, AH8-7 of Hap_3, DQS9-1 of Hap_12 and BKY-2 of Hap_6, *Pi02860* effector was expressed mostly at 10°C, followed by at 18°C. The effector was least expressed at 25°C. At 18°C, the nearly optimum infection temperature of the pathogen, the expression of *Pi02860* gene in seven isolates was upregulated >3 times with the highest upregulation of ~20 times in isolate BHYW-9 of Hap_1 whereas the expression in other isolates was <2 times (Figure 8). At 10°C, seven isolates were upregulated for ~20 times with the most upregulation of >300 times in isolate CHZ-62 of Hap_11. At 25°C, only three isolates were upregulated >2 times.

## Discussion

Due to their critical roles in host-pathogen interactions, pathogen effectors are important proteins regulating disease initiation and epidemics in natural and agricultural ecosystems (Uhse and Djamei, 2018). Population genetic analysis of effector genes would provide useful insights on how these important proteins evolve and what ecological functions they play. In addition to their roles in regulating host pathogen interaction, it has been documented that effector genes also have other ecological functions such as antimicrobial activities (Snelders et al., 2020). To maintain these multifaceted functions, effector genes are expected to have a higher evolvability equipped by high genetic variation for quick adaptation to constantly changing biotic and abiotic environments. Indeed, previous studies have reported that the genetic variation in effector genes is higher than many other functional genes in *P. infestans* and other pathogen species, possibly due to a synergic effect of recombination (Yang et al., 2018; Dale et al., 2019), plasticity (Timilsina et al., 2020) and multiple mutation mechanisms including pseudogenization, point mutation, deletion, insertion and truncation (Shen et al., 2021; Waheed et al., 2021). The physical locations of many effector genes in the sparse regions of genomes enriching with transposable elements provide unique opportunity for mutations to occur with a minimum fitness penalty to pathogen species (Haas et al., 2009; Dong et al., 2015). In this study, we found >20 nucleotide haplotypes in ~350 *Pi02860* sequences analysed. This is much higher than the genetic variation of other functional genes in this species. For example, we only identified < 10 nucleotide haplotypes in the 165 eEF1a sequences (Wang et al., 2021b) and two nucleotide haplotypes in the 140 ATP6 sequences (Zhang et al., 2017). However, the genetic variation found in *Pi02860* is much lower than other effector genes of the pathogen such as *Avr1, Avr2*, *Avr3a* and *Avr4* (Yang et al., 2018, 2020; Shen et al., 2021; Waheed et al., 2021). In addition, only point mutation and deletion were found in the current study. On the other hand, many variation mechanisms including pseudogenization, point mutation, deletion, insertion and truncation were found in *Avr1*, *Avr2*, *Avr3a* and *Avr4* of *P. infestans.*

These differences in genetic variation and mutation mechanisms between the *Pi02860* and other effector genes of *P. infestans* may reflect differences in interactions with host resistance genes. The corresponding resistance genes of effectors *Avr1*, *Avr2*, *Avr3a* and *Avr4* in our previous publications have been used commercially over a wide spatial scale in the past decades. Due to the constant interaction with host immunity systems, it is expected that this group of effector genes have become equipped with higher genetic variation to maintain the pathogen’s co-evolutionary pace with the host. On the other hand, *Pi02860* is only a candidate effector gene with some documented functions (Yang et al., 2016b; He et al., 2018) of regulating host immunity responses. No corresponding resistance gene has been identified in the potato host or has been used commercially. In this case, standing genetic variation in the effector gene is not essential for reproduction and transmission of the pathogen and therefore would be gradually purged out either due to random genetic drift (Lynch et al., 2016) or genetic load (Kirkpatrick and Jarne, 2000) incurred to the pathogen in its presence. It is documented that plant pathogens have optimized their effector reservoirs over their co-evolution journey with their hosts. Some effectors play a more important and common role in pathogen virulence than others. This group of effectors are more conserved in sequence structure and are regard as core effectors (Chepsergon et al., 2021). It is possible that *Pi02860* is also one of such core effectors.

We found mutations in the effector domain of *Pi02860* did not change its subcellular localization. The effectors were only found in the nucleus and cytoplasm, confirming that *Pi02860* is a cytoplasmic effector (Yang et al., 2016b). However, mutations in this region dramatically affect the fitness of the pathogen as indicated by the statistical difference among the nucleotide haplotypes in their ability to suppress the host’s immunity response and the amount of disease they produced (Figures 5, 7). Overall, these mutations reduce the ability of the pathogen to inhibit host immunity responses, leading to less disease even though the positive correlation between the two parameters is not significant (Figure 6). This reduction in immunity suppression is more obvious for the haplotype with the C-terminal truncation (Hap_8), further suggesting that *Pi02860* may be a core effector which is crucial for *P. infestans* pathogenicity and its loss from the genome may lead to a severe fitness penalty to the pathogen.

In addition to host resistance, other ecological factors may also contribute to the evolution of effector genes. Previously, it was reported that microbial community structure in the plant root rhizosphere can affect the evolutionary outcome of effector genes in a plant pathogen (Bever et al., 2012). Here, we found that abiotic factors may also contribute to the evolution of the effector gene in *P. infestans*. Genetic variation of *Pi02860* in terms of haplotype richnesss, haplotype diversity and nucleotide diversity increased as the altitude of the sample collection sites increased (Figures 2B,C, 3A). This spatial pattern of genetic variation is also linked to the fitness of the pathogen (Figure 6). Mutation rate, particularly the synonymous mutation rate, also increased as the altitude of the collection site increased (Figures 3D–F). Because no resistance gene corresponding to *Pi02860* was used in the fields and the isolates included in the study were sampled from the same potato variety on the same day at the same region, the observed difference in the genetic variation among altitudinal *Pi02860* effector is unlikely to have been caused by natural selection for host adaptation. Rather, it more likely reflects an elevated mutation rate caused by the vertical distribution of climatic conditions such as UV radiation and temperature. UV radiation is a major mutagenic agent which promotes the generation of genetic variation in species by inducing structural change of genetic material in the genome. For example, the mutation rate in bacteria increased hundreds of times after exposed to UV radiation (Shibai et al., 2017). In clear skies, UV radiation increases by 10–20% in response to a 1,000 m increase in altitude (Blumthaler et al., 1992; Schmucki and Philipona, 2002; Narayanan et al., 2010).

Temperature is an important environmental factor with crucial impacts on nearly all biological and evolutionary processes of species (Townsley and Yildiz, 2015; Huot et al., 2017; Garbelotto et al., 2021). Recently, it was reported that temperature can regulate the population genetic structure and evolution of effector genes (Velásquez et al., 2018; Waheed et al., 2021). In the current study, we found the expression of *Pi02860* was regulated by experimental temperatures. The effector gene demonstrated a higher expression level at a lower temperature (10°C) compared to the near optimum infection temperature of the pathogen (~19°C), and the expression level was further reduced as the experimental temperature increased to 25°C (Figure 8). Air temperature at the earth surface is negatively correlated with altitude (Legates and Willmott, 1990) and it is possible that changes in temperature may also contribute to the observed association of genetic variation in *Pi02860* with altitude. Recombination increases genetic variation of species by reshuffling genetic material among genomes and reducing the purging effect of natural selection (Gossmann et al., 2014). Both UV radiation and temperature can affect the recombination rate of species (Boyko et al., 2005; Shibai et al., 2017). Recombination events regulated by vertical distributions of temperature may also contribute to the observed association of genetic variation with altitude, but this hypothesis is rejected by the association analysis between recombination rate and the altitudes of collection sites. We found neither putative recombination events by the seven algorithms (RDP, GENECONV, Bootscan, MaxChi, Chimaera, SiScan and 3Seq) embedded in the RDP4 suite (Martin et al., 2015) nor association between recombination rate and altitude. However, intragenic recombination has been documented in several other effector genes of the pathogen sampled from the region (Yang et al., 2020; Shen et al., 2021; Waheed et al., 2021).

Many genetic and ecological factors influence the generation and maintenance of genetic variation. To minimize this effect, we sampled from the same potato variety along a hill on the same day. Our results indicate that in additional to host, climatic conditions associated with altitudinal distribution also contribute to the evolution of effector gene *Pi02860*. It has to point out that only one effector was reported in the current manuscript, further studies involving more effector genes and pathogen species are needed to confirm the altitudinal pattern. However, our results are complementary to other evolutionary phenomena that also show altitudinal effect on pathogen evolution at both the genic and organismic level (Santini and Ghelardini, 2015; Wang et al., 2021a). Further studies involving more effector genes are needed and may therefore, have many important implications for future agricultural and ecological sustainability caused by ongoing climate changes.

## Data availability statement

The *Pi02860* haplotype sequence data presented in the study has been deposited in NCBI. The GenBank accession numbers of hap_1, 2, 3, 4, 6, 9, 10, 11, 12, 13, 14, 15, 16, 17, 18, 19, 20, 21 are OP222576, OP222577, OP222578, OP222579, OP222580, OP222581, OP222582, OP222583, OP222584, OP222585, OP222586, OP222587, OP222588, OP222589, OP222590, OP222591, OP222592, and OP222593. The GenBank accession numbers of hap_5, 7, 8 are OP222594, OP222595, and OP222596.

## Author contributions

L-NY and HO performed the experiments, analysed data and wrote the manuscript. ON, AW, and Y-PW collected pathogen isolates. HF and WL genotyped pathogen isolates. JZ conceived, designed and supervised the experiments, analysed the data and wrote the manuscript. All authors contributed the article and approved the submitted version.

## Funding

This work was supported by the National Natural Science Foundation of China (grant no. 31901861).

## Conflict of interest

The authors declare that the research was conducted in the absence of any commercial or financial relationships that could be construed as a potential conflict of interest.

The reviewer RV declared a shared affiliation with one of the authors JZ to the handling editor at the time of review.

## Publisher’s note

All claims expressed in this article are solely those of the authors and do not necessarily represent those of their affiliated organizations, or those of the publisher, the editors and the reviewers. Any product that may be evaluated in this article, or claim that may be made by its manufacturer, is not guaranteed or endorsed by the publisher.
