# Supplementary material for: Genetic variation along an altitudinal gradient in the Phytophthora infestans effector gene Pi02860
Source: Front Microbiol. 2022 Sep 8;13:972928. doi: 10.3389/fmicb.2022.972928 (PMC9492930; doi:10.3389/fmicb.2022.972928)
Supplement: Supplementary file 1 [file Data_Sheet_1.docx]

**Supplementary Material**

**Supplementary Table 1** The positions and mutation types of the 21 nucleotide haplotypes of *Pi02860*. Dot represents the same nucleotide with Hap_1 and “-” represents single nucleotide deletion.

| Position | **1** | **2** | **3** | **4** | **5** | **18** | **22** | **30** | **55** | **107** | **110** | **115** | **140** | **143** | **154** | **158** | **174** | **191** | **199** | **214** | **219** | **222** | **226** | **239** | **254** | **257** | **258** | **267** | **269** | **286** | **295** | **299** | **344** | **362** | **376** |
| --- | --- | --- | --- | --- | --- | --- | --- | --- | --- | --- | --- | --- | --- | --- | --- | --- | --- | --- | --- | --- | --- | --- | --- | --- | --- | --- | --- | --- | --- | --- | --- | --- | --- | --- | --- |
| **Hap_1** | A | T | G | C | G | C | C | C | C | C | T | A | G | A | C | C | C | G | C | C | C | C | G | C | C | C | G | G | C | A | T | A | C | T | C |
| **Hap_2** | **.** | **.** | **.** | **.** | **.** | **.** | **.** | **.** | **.** | **.** | **.** | **.** | **.** | G | **.** | **.** | **.** | **.** | **.** | **.** | **.** | **.** | **.** | **.** | **.** | **.** | **.** | **.** | **.** | **.** | **.** | **.** | **.** | **.** | **.** |
| **Hap_3** | **.** | **.** | **.** | **.** | **.** | **.** | **.** | T | **.** | **.** | **.** | **.** | **.** | **.** | **.** | **.** | **.** | **.** | **.** | **.** | **.** | **.** | **.** | **.** | **.** | **.** | **.** | **.** | **.** | **.** | **.** | **.** | **.** | **.** | **.** |
| **Hap_4** | **.** | **.** | **.** | **.** | **.** | **.** | **.** | T | **.** | **.** | **.** | **.** | **.** | G | **.** | **.** | **.** | **.** | **.** | **.** | **.** | **.** | **.** | **.** | **.** | **.** | **.** | **.** | **.** | **.** | **.** | **.** | **.** | **.** | **.** |
| **Hap_5** | **-** | **.** | **.** | **.** | **.** | **.** | **.** | . | **.** | **.** | **.** | **.** | **.** | **.** | **.** | **.** | **.** | **.** | **.** | **.** | **.** | **.** | **.** | **.** | **.** | **.** | **.** | **.** | **.** | **.** | **.** | **.** | **.** | **.** | **.** |
| **Hap_6** | **.** | **.** | **.** | **.** | **.** | G | G | T | **.** | **.** | **.** | **.** | **.** | **.** | **.** | **.** | **.** | **.** | **.** | **.** | **.** | **.** | **.** | **.** | **.** | **.** | **.** | **.** | **.** | **.** | **.** | **.** | **.** | **.** | **.** |
| **Hap_7** | **-** | **-** | **-** | **-** | **-** | **.** | **.** | T | **.** | **.** | **.** | **.** | **.** | **.** | **.** | **.** | **.** | **.** | **.** | **.** | **.** | **.** | **.** | **.** | **.** | **.** | **.** | **.** | **.** | **.** | **.** | **.** | **.** | **.** | **.** |
| **Hap_8** | **.** | **.** | **.** | **.** | **.** | **.** | **.** | T | A | A | **.** | **.** | **.** | G | G | T | T | . | T | A | G | A | **.** | **.** | T | A | **.** | **.** | **.** | **.** | **.** | T | **.** | **.** | **.** |
| **Hap_9** | **.** | **.** | **.** | **.** | **.** | **.** | **.** | T | **.** | **.** | **.** | **.** | **.** | G | **.** | **.** | **.** | **.** | **.** | **.** | **.** | **.** | **.** | **.** | **.** | **.** | **.** | **.** | **.** | **.** | **.** | **.** | **.** | **.** | T |
| **Hap_10** | **.** | **.** | **.** | **.** | **.** | **.** | **.** | T | **.** | **.** | **.** | **.** | **.** | G | **.** | **.** | **.** | **.** | **.** | **.** | **.** | **.** | T | **.** | **.** | **.** | **.** | **.** | **.** | **.** | **.** | **.** | **.** | **.** | **.** |
| **Hap_11** | **.** | **.** | **.** | **.** | **.** | **.** | **.** | T | **.** | **.** | **.** | **.** | **.** | G | **.** | **.** | **.** | **.** | **.** | **.** | **.** | **.** | **.** | **.** | **.** | **.** | **.** | **.** | T | **.** | **.** | **.** | **.** | **.** | **.** |
| **Hap_12** | **.** | **.** | **.** | **.** | **.** | **.** | **.** | T | **.** | **.** | **.** | **.** | **.** | G | **.** | **.** | **.** | **.** | **.** | **.** | **.** | **.** | **.** | **.** | **.** | **.** | **.** | **.** | **.** | G | **.** | **.** | **.** | **.** | **.** |
| **Hap_13** | **.** | **.** | **.** | **.** | **.** | **.** | **.** | T | **.** | **.** | **.** | C | **.** | G | **.** | **.** | **.** | **.** | **.** | **.** | **.** | **.** | **.** | **.** | **.** | **.** | **.** | **.** | **.** | **.** | **.** | **.** | **.** | **.** | **.** |
| **Hap_14** | **.** | **.** | **.** | **.** | **.** | **.** | **.** | T | **.** | **.** | **.** | **.** | **.** | G | **.** | **.** | **.** | **.** | **.** | **.** | **.** | **.** | **.** | **.** | **.** | **.** | **.** | **.** | **.** | **.** | **.** | **.** | **.** | C | **.** |
| **Hap_15** | **.** | **.** | **.** | **.** | **.** | **.** | **.** | T | **.** | **.** | **.** | **.** | **.** | G | **.** | **.** | **.** | **.** | **.** | **.** | **.** | **.** | **.** | T | **.** | **.** | **.** | **.** | **.** | **.** | **.** | **.** | **.** | **.** | **.** |
| **Hap_16** | **.** | **.** | **.** | **.** | **.** | **.** | **.** | T | **.** | **.** | **.** | **.** | **.** | **.** | **.** | **.** | **.** | **.** | **.** | **.** | **.** | **.** | **.** | **.** | **.** | **.** | **.** | T | **.** | **.** | C | **.** | T | **.** | **.** |
| **Hap_17** | **.** | **.** | **.** | **.** | **.** | **.** | **.** | T | **.** | **.** | **.** | **.** | **.** | G | **.** | **.** | **.** | **.** | **.** | **.** | **.** | **.** | **.** | **.** | **.** | **.** | A | **.** | **.** | **.** | C | **.** | **.** | **.** | **.** |
| **Hap_18** | **.** | **.** | **.** | **.** | **.** | **.** | **.** | T | **.** | **.** | **.** | **.** | A | **.** | **.** | **.** | **.** | **.** | **.** | **.** | **.** | **.** | **.** | **.** | **.** | **.** | **.** | **.** | **.** | **.** | C | **.** | **.** | **.** | **.** |
| **Hap_19** | **.** | **.** | **.** | **.** | **.** | **.** | **.** | T | **.** | **.** | C | **.** | **.** | G | **.** | **.** | **.** | **.** | **.** | **.** | **.** | **.** | **.** | **.** | **.** | **.** | **.** | **.** | **.** | **.** | **.** | **.** | **.** | **.** | **.** |
| **Hap_20** | **.** | **.** | **.** | **.** | **.** | **.** | **.** | T | **.** | **.** | **.** | **.** | **.** | **.** | **.** | **.** | **.** | **.** | **.** | **.** | **.** | **.** | **.** | **.** | **.** | **.** | **.** | **.** | **.** | **.** | C | **.** | **.** | **.** | **.** |
| **Hap_21** | **.** | **.** | **.** | **.** | **.** | **.** | **.** | T | **.** | **.** | **.** | **.** | **.** | **.** | **.** | **.** | **.** | A | **.** | **.** | **.** | **.** | **.** | **.** | **.** | **.** | **.** | **.** | **.** | **.** | C | **.** | **.** | **.** | **.** |

**Supplementary Table 2** The frequency distribution of *Pi02860* nucleotide haplotypes in each of five populations. Haplotypes detected only once are combined into others.

|  | **A** | **B** | **C** | **D** | **E** | **Total** |
| --- | --- | --- | --- | --- | --- | --- |
| **Hap_1** | 60.00 | 62.79 | 45.76 | 49.35 | 50.75 | 54.24 |
| **Hap_2** | 23.08 | 19.77 | 27.12 | 28.57 | 22.39 | 24.01 |
| **Hap_3** | 10.77 | 15.12 | 22.03 | 10.39 | 16.42 | 14.69 |
| **Hap_4** | 4.62 | 1.16 | 0.00 | 3.90 | 1.49 | 2.26 |
| **Others** | 1.54 | 1.16 | 5.08 | 7.79 | 8.96 | 4.80 |
| **Sum** | 100 | 100 | 100 | 100 | 100 | 100 |

**Supplementary Table 3** The frequency distribution of *Pi02860* amino acid haplotypes in each of the five populations. Hap_1 and Hap_3 are translated into the same isoform, Hap_2 and Hap_4 are translated into the same isoform. Haplotypes detected only once are combined into others.

|  | **A** | **B** | **C** | **D** | **E** | **Total** |
| --- | --- | --- | --- | --- | --- | --- |
| **Hap_1&3** | 70.77 | 77.91 | 67.80 | 59.74 | 67.16 | 68.93 |
| **Hap_2&4** | 27.69 | 20.93 | 27.12 | 32.47 | 23.88 | 26.27 |
| **Others** | 1.54 | 1.16 | 5.08 | 7.79 | 8.96 | 4.80 |
| **Sum** | 100 | 100 | 100 | 100 | 100 | 100 |
